# The Detection of Sea Buckthorn Juice SSC Based on a Portable Near-Infrared Spectrometer Combined with an MoE-CNN Prediction Model

**DOI:** 10.3390/foods15010144

**Published:** 2026-01-02

**Authors:** Hao Niu, Yabo Zhang, Shiqi Hu, Hong Zhang, Yang Liu

**Affiliations:** 1 College of Mechanical and Electrical Engineering, Tarim University, Alar 843300, China; 2 Modern Agricultural Engineering Key Laboratory at Universities of Education Department of Xinjiang Uygur Autonomous Region, Alar 843300, China; 3 Xinjiang Production and Construction Corps Key Laboratory of Utilization and Equipment of Special Agricultural and Forestry Products in Southern Xinjiang, Alar 843300, China; 4 College of Animal Science, Tarim University, Alar 843300, China

**Keywords:** sea buckthorn, near-infrared spectrometer, soluble solids, mixture of experts convolutional neural network

## Abstract

The use of a portable near-infrared (NIR) spectrometer for detecting sea buckthorn juice SSC has not been explored. In this study, spectral data of 180 juice samples were collected using a portable NIR spectrometer. An SSC prediction model based on a mixture of experts convolutional neural network (MoE-CNN) was proposed. The MoE-CNN model was compared with traditional chemometric models in terms of prediction performance and feature extraction capability. The results showed that detecting the SSC of sea buckthorn juice using a portable NIR spectrometer combined with the MoE-CNN model is feasible. The optimal chemometric model, CARS-PLS, achieved RMSEP and RPD values of 1.42% and 2.67, respectively. The MoE-CNN model outperformed chemometric models and the CNN model, achieving an RMSEP of 1.26% and RPD of 3.02. Compared with CARS-PLSR, MoE-CNN adaptively weighted spectral features through MoE and feature fusion modules, effectively suppressing spectral noise and improving detailed feature extraction. These findings demonstrate that combining a portable NIR spectrometer with MoE-CNN is effective for rapid SSC detection in sea buckthorn juice. This study provides a new approach for the rapid detection of sea buckthorn juice SSC.

## 1. Introduction

Sea buckthorn juice possesses extremely high nutritional value [1] which is rich in vitamin C, organic acids, and various trace elements. Rapid and accurate determination of the component content in sea buckthorn juice is crucial for quality control and production process optimization. The soluble solids content (SSC) is an important indicator for evaluating juice quality. SSC comprehensively reflects the flavor characteristics and sea buckthorn juice sweetness level, directly affecting the product’s taste experience and market value [2] (Leahu et al., 2022). Therefore, achieving rapid and accurate detection of sea buckthorn juice SSC is significant for product quality assessment and market pricing. Currently, sea buckthorn juice SSC detection usually relies on handheld or laboratory refractometers. Handheld refractometers are easy to operate, but their detection accuracy is relatively low. The results are easily affected by the operating and sample turbidity, which leads to instability. Laboratory refractometers offer higher accuracy, but the equipment is expensive and bulky. In addition, samples usually require complex pretreatment before measurement, making them unsuitable for rapid, online, and large-scale detecting [3] (Jaywant et al., 2022). Therefore, developing a rapid and efficient detection method for evaluating sea buckthorn juice SSC is important.

Near-infrared (NIR) spectroscopy, as a rapid and high-precision online detection technique, has been widely applied in the quality assessment of liquid foods, including pineapple juice [4] (Lamptey et al., 2024), blueberry juice [5] (Yang et al., 2024), beetroot juice [6] (Bahrami et al., 2020), sugarcane juice [7] (Njume et al., 2025), and orange juice [8] (Miro et al., 2025). NIR spectroscopy can reflect the vibrational characteristics of components at specific wavelength bands, providing a reliable method for quantitative analysis of key components in liquid foods [9] (Zhao et al., 2024). Therefore, NIR spectroscopy has potential for detecting sea buckthorn juice SSC. However, current studies mostly rely on large benchtop spectrometers, which offer high detection accuracy but are difficult to apply for real-time sample analysis on the production lines or in the field. In contrast, portable NIR spectrometers are small in size, low in cost, and convenient for rapid on-site analysis, providing a new technical approach for fast quality assessment of liquid foods [10] (Yang et al., 2021). However, portable spectrometers also have limitations, such as a lower signal-to-noise ratio and sensitivity to environmental variations. Therefore, it is necessary to develop an efficient prediction model that is capable of suppressing spectral noise, enhancing signal stability, and extracting features highly correlated with the target. The literature review indicates that no studies have explored the feasibility of quantitatively detecting sea buckthorn juice SSC using portable NIR spectrometers.

NIR spectroscopy is commonly combined with chemometric methods [11] (Li et al., 2025) or deep learning networks [12] (Deng et al., 2024) to model the relationship between spectral data and target component content [13] (Lun et al., 2025). Traditional chemometric methods typically involve preprocessing, feature extraction, and target regression. Selecting appropriate preprocessing, feature extraction, and regression algorithms is crucial for achieving high accuracy and robustness. However, selecting these algorithms largely relies on the operator’s experience and skills [14] (Chen et al., 2025). This process is time-consuming and cumbersome and carries the risk of omitting key features. In recent years, deep learning has made significant progress in data analysis. Convolutional neural networks (CNNs) have become an efficient and popular tool for feature extraction and model construction. The combination of CNNs and spectroscopic techniques has been successfully applied in the detection of food components [12] (Deng et al., 2024). In conclusion, deep learning and traditional chemometric methods have been applied to detect component content in various liquid foods. However, the application of deep learning and traditional chemometric methods for the quantitative detection of sea buckthorn juice SSC remains limited. Therefore, this study aims to compare the predictive performance of sea buckthorn juice SSC using a portable NIR spectrometer combined with deep learning networks and traditional chemometric models. At the same time, the best method for using a portable near-infrared spectrometer to detect SSC was explored.

The specific objectives of this study are as follows: (1) to analyze the spectral characteristics and physicochemical properties of sea buckthorn juice; (2) to construct a traditional chemometric model predicting sea buckthorn juice SSC; (3) to propose a mixture of experts-based convolutional neural network (MoE-CNN) model predicting sea buckthorn juice SSC; (4) to compare the predictive performance of the traditional chemometric models and MoE-CNN model; and (5) to perform an interpretability analysis of the two types of models to reveal their differences in spectral feature extraction.

## 2. Materials and Methods

### 2.1. Sea Buckthorn Juice Samples

To obtain sea buckthorn juice samples of different qualities and improve the representativeness of the samples, this study collected products from major producing regions in China, including Xinjiang, Gansu, Shanxi, and Qinghai. The samples (180) covered different brands and various concentrations of original pulp. During transportation, all samples were kept below 10 °C, and upon arrival at the laboratory, they were immediately stored in a 4 °C constant-temperature refrigerator. Prior to quality parameter assessment and spectral measurements, all samples were kept at room temperature (25 ± 1 °C) for 12 h to minimize measurement errors due to temperature fluctuations.

### 2.2. NIR Acquisition of Sea Buckthorn Juice

**Figure 1:**
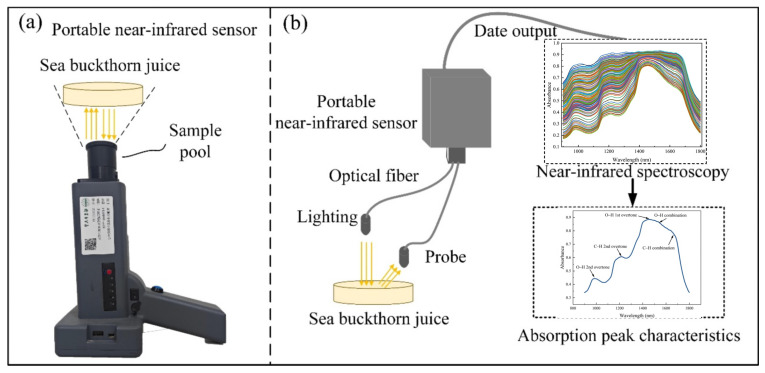
(**a**) Handheld near-infrared spectrometer, (**b**) schematic diagram of near-infrared spectral measurement.

In this study, the NIR spectra of sea buckthorn juice samples were measured by a handheld NIR spectrometer (NIRmagic3500, Beijing Weichuangyingtu Technology Co., Ltd., Beijing, China), as shown in Figure 1a. Before measurement, all samples were thoroughly mixed in a shaker to guarantee homogeneity and consistent spectral measurements. Near-infrared spectroscopy was performed using diffuse reflectance mode. The mixed sea buckthorn juice was placed in a dedicated liquid sample pool, and the absorbance of the sea buckthorn juice between 900 and 1800 nm was collected using a spectrometer, as shown in Figure 1b. The sampling interval was 1 nm, the spectral resolution was less than 16 nm, and the measurement time for a single scan was less than 5 s. To ensure data reproducibility and reliability, each sample was measured three times, and the average spectrum was used as the final spectral data. Spectral measurements were conducted in the Key Laboratory of Modern Agricultural Engineering at Tarim University (Xinjiang, China), with the laboratory temperature maintained at 25 ± 1 °C to simulate natural environmental conditions in Xinjiang.

### 2.3. Sea Buckthorn Juice SSC Detection

In this study, the SSC of sea buckthorn juice was measured using a DigiPol-R300 refractometer (Jiahang, Shanghai, China). Prior to analysis, all 180 sea buckthorn juice samples were thoroughly vortexed. Two samples containing trace amounts of solid material were then subjected to sieve filtration. Following pretreatment, the soluble solids content of all samples was measured using a refractometer. Briefly, two drops of the pretreated juice were pipetted onto the center of the refractometer prism for measurement. After each measurement, the prism was rinsed with ultrapure water and wiped with lens tissue to ensure no residue remained before measuring the next sample. Each sample was tested twice, with each test repeated three times, and the average value was taken as the SSC measurement result for the sample.

### 2.4. Establishment of the Prediction Models

#### 2.4.1. Abnormal Sample Removal and Sample Segmentation

To remove abnormal samples caused by instrument errors and environmental variations in spectral data, this study used Principal Component Analysis (PCA) and the Isolation Forest (IF) algorithm to identify and eliminate abnormal samples. PCA was employed to reduce the dimensionality of high-dimensional spectral data, extracting the main features and minimizing noise interference. Subsequently, the IF algorithm was used to remove abnormal samples in order to improve data quality. After removing abnormal samples, the dataset was partitioned using the SPXY (Sample set partitioning based on joint X–Y distances) method to ensure scientific and representative sampling. The filtered samples were divided into a calibration set (70%) for model development and a prediction set (30%) for model evaluation. The SPXY method, which considers the distributions of both independent (X) and dependent (Y) variables, ensures that the calibration and prediction set uniformly cover the feature space, thereby improving the rationality of model training and validation.

#### 2.4.2. Construction of the MoE-CNN Model

**Figure 2:**
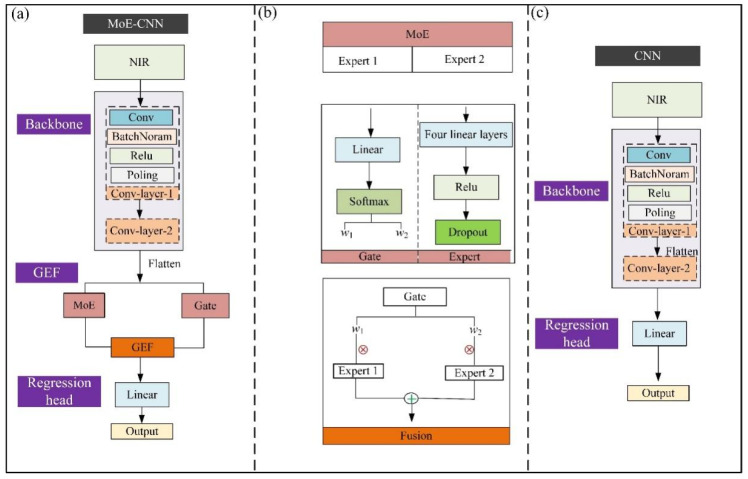
(**a**) Structure of the MoE-CNN model, (**b**) structure of the GEF module, and (**c**) structure of the CNN model. Note: ⊕ represent add; ⨂ represent multiplication.

To construct a high-accuracy prediction model for soluble solids content (SSC), this study was inspired by the visual attention mechanism and designed a convolutional neural network model based on a mixture of experts (MoE-CNN). The model primarily consists of three components: a feature extraction module (Backbone), a Gating-Expert Fusion (GEF) module, and a regression head, as illustrated in Figure 2a. The backbone contains two convolutional layers, each layer performing convolution, batch normalization (BN), ReLU activation, and pooling operations sequentially to progressively extract both local and global spectral features. Convolutional layers are commonly included in the GEF module. However, preliminary experiments revealed that introducing convolutional layers into the GEF module tends to cause overfitting. This phenomenon may arise from the limited feature dimensionality of one-dimensional spectral data, where an excessively deep convolutional network can easily overfit. Therefore, the GEF module consists solely of a mixture of experts (MoE) unit (including two experts), a Gating unit, and a Fusion unit, as shown in Figure 2b, which perform adaptive weighted fusion of the input features. Finally, the regression head is composed of a single linear layer that maps the fused features to the target output.

The near-infrared spectra were first processed by the feature extraction module and then flattened before being separately fed into the Gating unit and the MoE unit. The Gating unit adaptively generates weights based on the distribution of input features to regulate the contribution of each expert’s output. Each expert subnetwork within the MoE unit independently learns distinct subspace distributions of spectral features, thereby capturing multi-scale and nonlinear information [15] (Rasti et al., 2019). Subsequently, the Fusion unit performs weighted integration of the expert outputs, enabling adaptive modeling of complex spectral representations. The formula of the Gating-Expert Fusion module is as follows:

(1) $$f_{fusion}=\overset{N}{\underset{i=1}{\sum }}w_{i}E_{i}$$

where *i* is the number of experts, *w_i_* represents the weight coefficient generated by the gating network, and *f_fusion_* denotes the fused features. Finally, the fused features are passed through the regression head to output the predicted target value. After pretraining the model, the key parameter settings of the MoE-CNN model are listed in Table 1.

To evaluate the effect of the GEF module on prediction performance, an ablation experiment was conducted by removing the GEF module from the MoE-CNN model while keeping all other components unchanged, as illustrated in Figure 2c. By comparing the performance of the MoE-CNN and CNN models on the same dataset, the contribution of the GEF module to the overall architecture was quantitatively assessed. All models were trained using the Adam optimizer with a learning rate of 0.001, and the mean squared error (MSE) was employed as the loss function.

### 2.5. Construction of Models Based on Chemometric Methods

The chemometric modeling process generally involves four main steps: data input, preprocessing, feature extraction, and target regression. In this study, the near-infrared spectra of sea buckthorn juice were used as input data. Spectral preprocessing was performed using the Savitzky–Golay (SG) smoothing algorithm to reduce noise. Feature extraction algorithms include Uninformative Variable Elimination (UVE), Variable Importance in Projection (VIP), Competitive Adaptive Reweighted Sampling (CARS), and the full-spectrum method (FS, without feature selection). UVE eliminates redundant wavelengths by introducing random noise variables as references to evaluate the stability of regression coefficients, removing those with significance below the noise threshold. VIP identifies key variables based on the weight structure of the Partial Least Squares Regression model, retaining those with VIP values greater than 1. The CARS algorithm dynamically selects optimal wavelength subsets that minimize the root mean square error of cross-validation through a combination of Monte Carlo sampling (randomly selecting 80–90% samples as the calibration set in each iteration), an exponential decay function (gradually eliminating weak variables), and adaptive reweighted sampling (adjusting the retention probability according to regression coefficient magnitude). In this study, 100 Monte Carlo iterations and 5-fold cross-validation were performed to ensure model robustness, enabling efficient purification of spectral features [11,16] (Li et al., 2025; Li et al., 2024). Target regression algorithms include Partial Least Squares Regression (PLSR), Support Vector Regression (SVR), and Least Squares Support Vector Machine (LSSVM). By comparing the predictive performance of models combining different feature extraction and regression algorithms, the optimal chemometric model was identified.

### 2.6. Predictive Performance Evaluation

In this study, the correlation coefficient (*R*), root mean square error (*RMSE*), and mean absolute error (MAE) were used to evaluate model accuracy. Additionally, relative predictive deviation (RPD) was employed to assess the predictive performance and robustness of the models. A model with RPD > 2.5 is considered to have good predictive accuracy and reliability [17] (Guo et al., 2024). The formulas for these metrics are as follows:

(2) $$R=\frac{\sum_{n}^{i=1}(Y_{i}-\bar{Y})(y_{i}-\bar{y})}{\sqrt{\sum_{n}^{i=1}{\left(Y_{i}-\bar{Y}\right)}^{2}}\sqrt{\sum_{n}^{i=1}{\left(y_{i}-\bar{y}\right)}^{2}}}$$

(3) $$RMSE=\sqrt{\frac{1}{n}\sum_{i=1}^{n}{\left(y_{i}-Y_{i}\right)}^{2}}$$

(4) $$RPD=\frac{SD_{V}}{RMSE}$$

where $y_{i}$ and $Y_{i}$ represent the measured and predicted SSC values of the *i*-th sample, respectively; $\bar{y}$ and $\bar{Y}$ are the measured mean value and the predicted mean value of SSC, respectively; n is the number of samples; and *SD_V_* and *RMSE* denote the standard deviation and root mean square error of SSC, respectively.

In addition, to further assess the reliability of the predictions from the optimized chemometric model and the MoE-CNN model on the prediction set, a paired t-test was performed between the predicted and measured values. This test assumes that the differences follow a normal distribution. The null hypothesis $H_{0}$ states that there is no systematic difference between the predicted and measured values (i.e., $\mu_{d}=0$). The test statistic is calculated as follows:

(5) $$t=\frac{\bar{d}}{s_{d}/\sqrt{n}}$$

where $d_{i}\;=\;Y_{i}\;-\;y_{i}$, $\bar{d}$ is the mean difference, $s_{d}$ is the standard deviation of the differences, and *n* is the number of samples. If the two-tailed test yields p >0.05, the null hypothesis is not rejected, indicating that no statistically significant systematic bias was detected between the predicted and measured values.

### 2.7. Interpretability Analysis of the CNN Model

To reveal the decision-making mechanism of the MoE-CNN model in predicting sea buckthorn juice SSC, this study employed the SHAP (Shapley Additive Explanations) method for interpretability analysis. The SHAP method is based on cooperative game theory. It quantifies feature importance by calculating and normalizing the contribution of each spectral feature value, enabling a quantitative assessment of each spectral band’s impact on the model output [18] (Zhang et al., 2025). Feature importance values were computed using the SHAP method via the Captum library in the PyTorch framework. These values were then visualized as importance maps, enabling the identification of key spectral bands that significantly influence the model’s predictions and revealing the contribution of each spectral feature to the outputs.

### 2.8. Software

In this study, the chemometrics algorithm was implemented in MATLAB 2024 (The Mathworks Inc., 1 Apple Hill Drive, Natick, MA, USA). The deep learning model ran on the PyTorch 1.7.1 framework and Python 3.7 platform. The computing equipment used was configured as follows: an Intel i7-12700H processor (Intel Corporation, 2200 Mission College Boulevard, Santa Clara, CA, USA), NVIDIA GeForce RTX 3060 Laptop GPU (6GB VRAM) (NVIDIA Corporation, 2788 San Tomas Expressway, Santa Clara, CA, USA), and 16GB RAM (2 × 8GB, DDR4) (Kingston Technology Company, Fountain Valley, CA, USA), equipped with NVIDIA CUDA 11.0 drivers (NVIDIA Corporation, 2788 San Tomas Expressway, Santa Clara, CA, USA), and the operating system was Windows 10 (Microsoft Corporation, One Microsoft Way, Redmond, WA, USA).

## 3. Results and Discussion

### 3.1. Statistical Analysis of Major Quality Parameters of Sea Buckthorn Juice

Table 2 presents the statistical results of the major quality parameters of the sea buckthorn juice samples. The results indicate that the ranges and coefficients of variation (CVs) for SSC are relatively large, suggesting significant differences among samples. These differences may be related to factors such as fruit variety, origin, maturity, and processing conditions. In summary, the SSC in the sea buckthorn juice samples shows considerable variation, indicating that the samples are representative.

### 3.2. Sea Buckthorn Juice Nir Spectral Analysis

**Figure 3:**
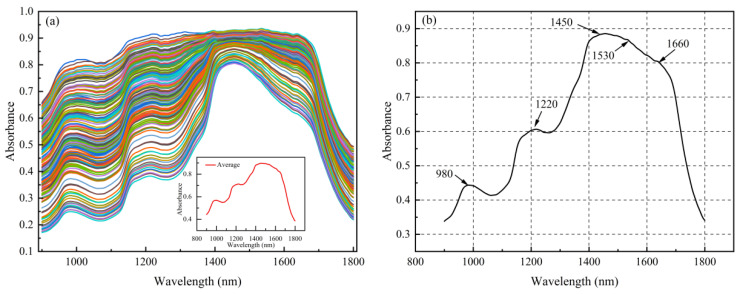
(**a**) NIR spectra of sea buckthorn juice samples and (**b**) absorption peaks.

Figure 3 shows the NIR absorption spectral features of the sea buckthorn juice samples. Figure 3a presents the spectra of all samples in the 900–1800 nm range. The spectral characteristics of all samples are generally consistent. However, differences in absorption intensity are observed among samples. This phenomenon reflects variation in the content of soluble solids and related components in the juice. Figure 3b shows the mean spectrum of the samples, with typical absorption peaks observed at approximately 980, 1220, 1450, 1530, and 1660 nm. The absorption peak at 980 nm is mainly attributed to the O–H stretching vibrations of water. The 1220 nm peak corresponds to C–H vibrations in sugars and organic acids, and the 1450 nm peak is related to the first overtone of water [19] (Fang et al., 2020). Peaks at 1530 and 1660 nm are associated with O–H or C–H vibrations [20] (Ehsani et al., 2023). The spectral features indicate that the SSC of sea buckthorn juice and its major components exhibit prominent absorption peaks in the NIR region, which provides a basis for constructing predictive models for SSC.

### 3.3. Removal of Abnormal Samples

**Figure 4:**
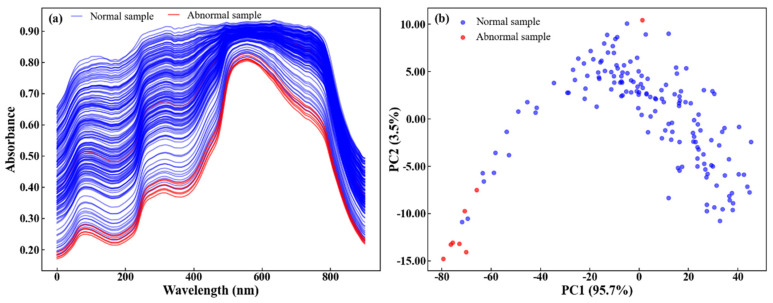
Normal and abnormal sample (**a**) spectra and (**b**) distribution.

Figure 4 shows the normal and abnormal sample spectra and distribution. The contributions of PC1 and PC2 were 95.7% and 3.5%, respectively, indicating that the first two principal components could explain most of the variance in the original spectral data. The PCA distribution results show that abnormal samples were mainly located at the edges of the scatter plot (the negative extreme region of PC1), while most normal samples were clustered together. Finally, a total of eight abnormal samples were removed from the dataset.

### 3.4. Prediction Model Performance Analysis

#### 3.4.1. Prediction Results

Table 3 presents the quantitative analysis results of different prediction models on the calibration set and prediction set. The results indicate that the MoE-CNN deep learning model shows significant advantages across all evaluation metrics, with RMSEP, Rp, and RPD values in the prediction set reaching 1.26%, 0.95, and 3.02, respectively. This result suggests that the MoE-CNN model exhibits high accuracy and stability in predicting sea buckthorn juice SSC. In contrast, the predictive performance of traditional chemometric models is significantly influenced by regression algorithms and feature extraction methods, resulting in noticeable differences in prediction results. Among them, the PLSR model combined with CARS achieved the best prediction results, with RMSEP, Rp, and RPD values in the prediction set of 1.42%, 0.93, and 2.67, respectively. However, its accuracy remains lower than the MoE-CNN model. This phenomenon suggests that the MoE-CNN model possesses stronger modeling capabilities in handling complex nonlinear relationships within spectral signals.

It is worth noting that the CARS-PLSR model relies on manual feature selection and repeated parameter adjustment, making the modeling process cumbersome and time-consuming. In contrast, the MoE-CNN features an end-to-end structure. It utilizes convolutional layers to automatically extract local and global spectral features and employs a gating mechanism to achieve weighted fusion of feature branches. This method requires no manual intervention and significantly improves the model’s generalization ability and prediction accuracy.

In the comparison of deep learning models, the MoE-CNN model outperformed the conventional CNN model in both prediction accuracy and generalization performance (RMSE: 1.49%, RPD: 2.55). It is noteworthy that the CNN model performed significantly better on the training set than on the prediction set, indicating a certain degree of overfitting. In contrast, the MoE-CNN model showed comparable RMSE values in the training and prediction sets, indicating strong generalization capability. This advantage is attributed to the GEF module. In this module, the contribution weights of each expert network are dynamically determined by the gating network, allowing adaptive fusion of multiple expert outputs. Unlike CNNs with a single feature extraction path, the MoE-CNN dynamically adjusts weight allocation according to spectral complexity. This adjustment enhances features relevant to the target variable and suppresses background noise. Moreover, the dynamic weighting strategy implemented by the gating mechanism enables the model to capture the nonlinear and multi-scale feature distributions of spectral data across different scales and hierarchical feature spaces.

In summary, the MoE-CNN model outperforms traditional chemometrics and standard CNNs in predicting sea buckthorn juice SSC, achieving highly reliable predictions.

#### 3.4.2. Comparative Analysis of Model Prediction Results

**Figure 5:**
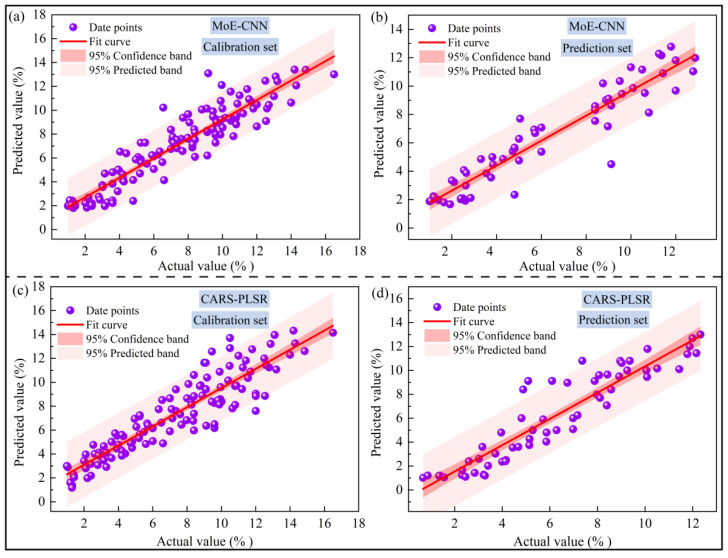
Fitting curves of SSC predicted values and measured values of the MoE-CNN model and CARS-PLSR model (solid lines) with 95% confidence and prediction bands: (**a**,**c**) calibration set, (**b**,**d**) prediction set.

Figure 5 shows the predicted versus actual values based on the MoE-CNN and CARS-PLSR models. The predictions for both the calibration and prediction sets agree well with the actual values, with most points lying near the fitting line and within the 95% confidence interval. The *p*-values of the paired t-test on the prediction set were 0.33 for the MoE-CNN and 0.81 for the CARS-LSSVM, both exceeding 0.05, indicating no statistically significant difference from the actual measurements. These results confirm that using a portable near-infrared spectrometer to detect sea buckthorn juice SSC is feasible. Moreover, the MoE-CNN outperforms CARS-PLSR in prediction accuracy, stability, and noise suppression. The expert hybrid architecture of the MoE-CNN can dynamically weight key spectral bands and suppress noise, ensuring robust performance. In contrast, CARS-PLSR relies on manual parameter tuning, which can easily lead to biased predictions and poor stability.

It should be noted that although both models perform well, their confidence intervals are relatively wide. This phenomenon can be explained by the raw data (Table 2). The sea buckthorn juice samples exhibit significant variability in SSC with a CV of 0.54, indicating substantial compositional differences. Large differences in component content between samples increase the prediction uncertainty of certain samples, resulting in wider confidence intervals. Therefore, to improve model accuracy and stability, future work needs to expand both the size and diversity of the sample set.

### 3.5. Interpretability Analysis of the Models

#### 3.5.1. Comparison of Feature Extraction Algorithms

**Figure 6:**
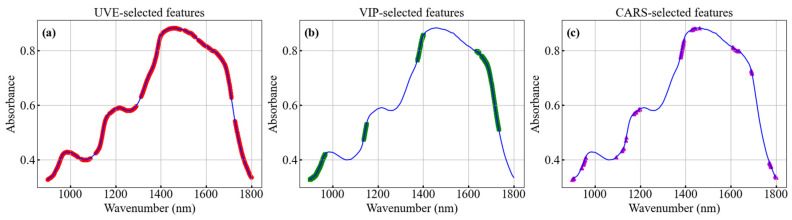
Feature bands selected by different feature extraction algorithms: (**a**) UVE, (**b**) VIP, (**c**) CARS.

Figure 6 shows the distributions of feature wavelengths extracted by the UVE, VIP, and CARS algorithms, respectively. The results indicate that UVE selects the largest number of wavelengths, covering almost the entire near-infrared range. VIP extracts a moderate number of wavelengths, which are relatively concentrated. CARS extracts the fewest wavelengths, mainly concentrated in the regions of 900–960 nm, 1380–1444 nm, and 1607–1637 nm. The CARS-PLSR model achieved the best SSC prediction performance, indicating that the wavelengths selected by CARS effectively captured the correlation between sea buckthorn juice SSC and the spectral information. In comparison, UVE retained excessive redundant and noisy variables, while VIP selected features similar to those of CARS but showed limited sensitivity to local nonlinear response regions (e.g., around 1200 nm). Overall, the stepwise competitive strategy of CARS preserved essential chemical information while reducing data redundancy, leading to superior model performance.

The above results indicate that different feature extraction algorithms exhibit distinct strengths and limitations, which directly influence the performance of regression models. This finding suggests that traditional chemometric approaches are highly dependent on the selection of feature extraction and regression algorithms.

#### 3.5.2. Interpretability Analysis of the CARS-PLSR Model

**Figure 7:**
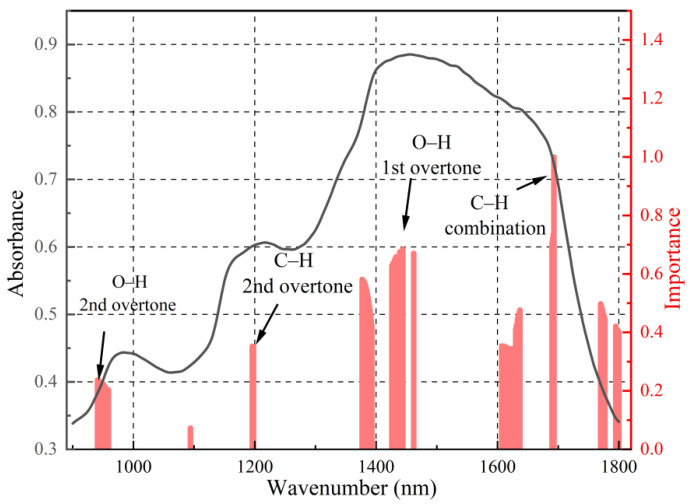
Distribution of key characteristic frequency bands of CARS-PLSR.

To reveal the decision-making mechanism of the SSC prediction model based on CARS feature selection and PLSR regression, the wavelengths selected by CARS were input into the PLSR model. The score and loading matrices were extracted through latent variable decomposition, and the key wavelengths were identified by averaging the absolute values of each wavelength’s loadings across all latent variables. The feature importance analysis (Figure 7) revealed that the major spectral weights were concentrated in near-infrared regions associated with O–H and C–H bond vibrations, including approximately 970 nm (second overtone of O–H stretching), 1200 nm (second overtone of C–H stretching), 1391 nm (combination of O–H bending and C–H stretching), 1450 nm (first overtone of O–H bending vibration), and 1650 nm (combination of C–H stretching and O–H bending). These characteristic bands correspond to typical vibration modes of hydroxyl and C–H bonds in water and sugar compounds [21,22] (Zhang et al., 2025; Munawar et al., 2022), consistent with the major chemical bonds of soluble solids.

#### 3.5.3. Interpretability Analysis of the Moe-Cnn Model

**Figure 8:**
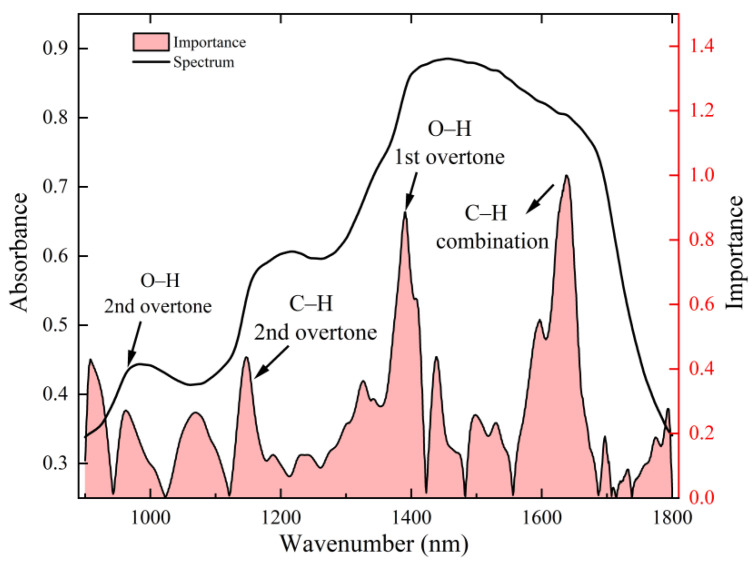
Distribution of key characteristic frequency bands of MoE-CNN.

The feature importance of the MoE-CNN model was analyzed using the SHAP method (Figure 8). The key spectral weights were primarily concentrated in near-infrared regions associated with O–H and C–H bond vibrations, including approximately 910 nm (O–H second overtone), 963 nm (C–H second overtone), 1070 nm (O–H and C–H combination bands), 1150 nm (C–H second overtone), 1337 nm and 1391 nm (O–H and C–H combination bands), 1450 nm (O–H first overtone), and 1650 nm (C–H combination bands). These characteristic wavelengths correspond to typical vibration modes of hydroxyl and C–H bonds in water and sugars [23,24], consistent with the chemical composition of soluble solids (SSC). The strong correspondence between spectral features and SSC-related chemical groups supports the reliability of MoE-CNN feature selection and the robustness of the model.

#### 3.5.4. Comparison of Model Feature Extraction

The performance of the CARS-PLSR and MoE-CNN models for near-infrared spectral detection of sea buckthorn juice SSC was compared. Both models extracted similar key spectral features, but the MoE-CNN achieved higher prediction accuracy and stability. Notably, CARS-PLSR missed significant peaks around 910 nm and 1070 nm, which are closely related to SSC. However, the MoE-CNN assigned higher weights to these regions. This indicates that the MoE-CNN has greater sensitivity to subtle signals.

This advantage arises from the mixture of experts (MoE) architecture. The Gating unit dynamically allocates expert weights according to the input spectrum, emphasizing relevant information while suppressing noise. Expert subnetworks capture complementary local and global features, which are then integrated by a Fusion unit. Consequently, MoE-CNN extracts fine spectral features more effectively from high-noise, high-dimensional data, enhancing robustness and generalization. These properties highlight its potential for rapid juice quality assessment.

### 3.6. Discussion

This study shows that a portable near-infrared spectrometer combined with an MoE-CNN model can detect SSC in sea buckthorn juice. The findings are discussed in the following two aspects.

#### 3.6.1. Application Potential of Nir Spectroscopy Combined with Moe-Cnn in Food Component Content Detection

NIR spectroscopy is a rapid, non-destructive technique widely used for detecting food components. However, predicting complex systems such as sea buckthorn juice is challenging due to spectral overlap, nonlinear effects, and noise from portable devices. Its high viscosity, abundant suspended solids, and high total solids further complicate spectral acquisition and modeling.

In this study, a portable NIR spectrometer was combined with an MoE-CNN model to predict SSC with high accuracy. The model’s gating-expert architecture enables adaptive fusion of multi-scale spectral features and effectively suppresses noise from low signal-to-noise ratios and sample variations. As a result, the MoE-CNN outperforms traditional chemometric models and standard CNNs in accuracy and robustness. With the development of portable spectrometers, this approach may allow rapid, low-cost quality detection in food production and consumer scenarios. It provides a promising tool for quality control in complex liquid foods.

#### 3.6.2. Comparative Analysis of Sea Buckthorn Juice Ssc Prediction Results

Quantitative prediction of sea buckthorn juice SSC using rapid detection techniques such as NIR spectroscopy is currently lacking. Therefore, this study compared the prediction results of sea buckthorn juice SSC with those reported in the literature for fruits and juices, such as apples and oranges. Compared with the results listed in latest review articles [9,25] (Zhao et al., 2024; Hemachandra et al., 2025), the RPD value (3.02) obtained in this study is close to or better than the prediction performance reported in some studies. It indicates that quantitative analysis of sea buckthorn juice SSC based on NIR spectroscopy is highly reliable. However, the RMSEP in this study is slightly higher than that reported for some other juices. This may be attributed to the high viscosity and strong light-scattering effect of sea buckthorn juice, which complicates NIR spectral signals and increases modeling errors. In addition, SSC showed relatively high coefficients of variation (Table 2), reflecting significant sample differences and further adding to modeling difficulty. Nevertheless, the MoE-CNN model achieved a high RPD, demonstrating accurate and stable predictions despite high sample variability. This suggests that a portable NIR spectrometer combined with an MoE-CNN can be applied for rapid quality detection of sea buckthorn juice. Future work should expand the sample size and diversity to improve representativeness and enhance the model’s generalization and prediction accuracy.

## 4. Conclusions

This study proposed a novel MoE-CNN model for predicting SSC in sea buckthorn juice. The differences in feature extraction and prediction performance between MoE-CNN and traditional chemometric models were evaluated. The feasibility of using a portable NIR spectrometer for SSC detection was explored. The results showed that both the MoE-CNN and CARS-PLSR achieved high prediction accuracy, demonstrating the application potential of portable spectrometers for detecting sea buckthorn juice SSC. The GEF module enables the CNN to adaptively weight and learn the distributions of complex spectral features. This enables suppression of irrelevant noise and accurate extraction of features strongly correlated with SSC. The optimal chemometric model, CARS-PLS, achieved RMSEP and RPD values of 1.42% and 2.67, respectively. The MoE-CNN model outperformed chemometric models and the CNN model, achieving an RMSEP of 1.26% and RPD of 3.02. Compared to the CARS-PLS model, the MoE-CNN is more effective at extracting detailed key features. This strategy enables the MoE-CNN to achieve excellent feature extraction and generalization on high-dimensional, nonlinear spectral signals. This approach provides a novel method for rapid, on-site detection of sea buckthorn juice SSC.

## Figures and Tables

**Table 1:** MoE-CNN model parameters.

**Layer**	**In Channels**	**Out Channels**	**Kernel Size**	**Stride**	**Padding**	**Output Shape**
Input	1	-	-	-	-	(1, 901)
Conv 1	1	2	5	1	2	(1, 901)
AvgPool	2	2	2	2	0	(1, 450)
Conv 2	2	4	3	1	1	(1, 450)
Flatten	4	1	-	-	-	(1, 1800)
Gate1	1800	2	-	-	-	(1, 2)
Expert 1	1800	600	-	-	-	(1, 600)
Expert 2	1800	600	-	-	-	(1, 600)
Regression head	600	1	-	-	-	(1, 1)

**Table 2:** Statistical results of major quality parameters of sea buckthorn juice.

Quality Properties	Max	Min	Average	SD	CV
SSC (%)	16.5	2.20	6.99	3.79	0.54

**Table 3:** Performance of the sea buckthorn juice SSC prediction models.

Models	Algorithms	Feature Extraction Algorithms	Calibration Set		Prediction Set		RPD
			RMSEC (%)	R_c_	RMSEP (%)	R_p_	
Chemometric models	GRNN	UVE	2.24	0.79	1.87	0.88	2.03
		VIP	1.56	0.90	2.14	0.82	1.78
		CARS	2.55	0.73	2.12	0.85	1.79
		FS	2.23	0.80	1.86	0.88	2.04
	LSVR	UVE	0.68	0.98	1.61	0.90	2.36
		VIP	1.41	0.92	1.63	0.91	2.33
		CARS	1.45	0.92	1.56	0.91	2.43
		FS	0.68	0.98	1.59	0.91	2.38
	PLSR	UVE	0.67	0.98	1.84	0.91	2.06
		VIP	1.53	0.91	1.46	0.92	2.60
		**CARS**	**1.57**	**0.90**	**1.42**	**0.93**	**2.67**
		FS	0.77	0.97	1.66	0.90	2.29
DNN models	**MoE-CNN**	**-**	**1.39**	**0.93**	**1.26%**	**0.95**	**3.02**
	**CNN**	-	**0.91**	**0.95**	**1.49**	**0.92**	**2.55**

## Data Availability

The original contributions presented in the study are included in the article; further inquiries can be directed to the corresponding author.
